# Delayed iatrogenic diaphragmatic hernia after thoracoscopic resection of diaphragm lipoma

**DOI:** 10.1186/s44215-023-00043-4

**Published:** 2023-06-08

**Authors:** Soichi Oka, Toshihiro Osaki, Teppei Hashimoto, Yuichiro Kawamura

**Affiliations:** 1 Chest Surgery, Kokura Memorial Hospital, Kitakyushu, 802-8555 Japan; 2 Gastroenterological Surgery, Kokura Memorial Hospital, Kitakyushu, Japan

**Keywords:** Diaphragmatic hernia, Lipoma, Thoracic surgery

## Abstract

**Background:**

Iatrogenic diaphragmatic hernias have been reported as a rare complication of thoracic and abdominal surgery. We herein report a case of delayed iatrogenic left diaphragmatic hernia after diaphragm pedunculated lipoma resection with minimally invasive surgery.

**Case presentation:**

A 72-year-old Japanese man was found to have an abnormal shadow by medical checkup X-ray and was admitted to our hospital. Chest computed tomography (CT) showed a 5 × 2-cm solid tumor at the left diaphragm. He was diagnosed with a left diaphragm tumor. We performed three-port video-assisted thoracic surgery. This tumor was pedunculated at the left central tendon of the diaphragm. We therefore dissected this tumor using an electric scalpel. Although there was about 5 × 4 mm in diameter slight heat damage to the diaphragm, it was not reinforced because it was very minor injury. He was diagnosed with a left diaphragmatic hernia without any symptoms by routine CT examination which scheduled 1 year after surgery. One day after hospitalization, on the morning of the operation, he suddenly complained of left back pain with acute exacerbation of the left diaphragmatic hernia. We therefore immediately performed emergency surgery and rescued this patient. No adverse events or complications were seen, and he was discharged on postoperative day 11. Three months after this surgery, this patient is doing very well.

**Conclusions:**

Caution should be exercised when using energy devices on the diaphragmatic surface, especially the left side, to avoid causing delayed diaphragmatic hernia. In cases of surgery involving the left-side diaphragm, it seems that careful follow-up after surgery is necessary.

## Background

Iatrogenic diaphragmatic hernias have been reported as a rare complication of thoracic and abdominal surgery [1–4]. Hashimoto et al. reported a rare case of delayed iatrogenic diaphragmatic hernia possibly due to diaphragmatic injury after soft coagulation for hemostasis during excision of peritoneal nodules on the left diaphragmatic surface to check for dissemination [5]. Several reports have suggested considering the possibility of delayed presentation of diaphragmatic hernia following thoracic and abdominal surgery involving an invasive technique with the diaphragm [1–3].

We herein report a case of delayed iatrogenic left diaphragmatic hernia after diaphragm pedunculated lipoma resection with minimally invasive surgery.

## Case presentation

A 72-year-old Japanese man was found to have an abnormal shadow by medical checkup X-ray and was admitted to our hospital. He had no symptoms and no remarkable medical, social, environmental, obstetrical, family, or employment history. He had smoked tobacco, and he occasionally drank alcohol. His blood pressure was 135/80 mmHg, his pulse was 80 bpm, and his body temperature was 36.5 °C. He had no significant abnormal findings on physical or neurological examinations. The blood cell count, liver and renal functions, and urine analysis findings were within normal limits.

**Fig. 1:**
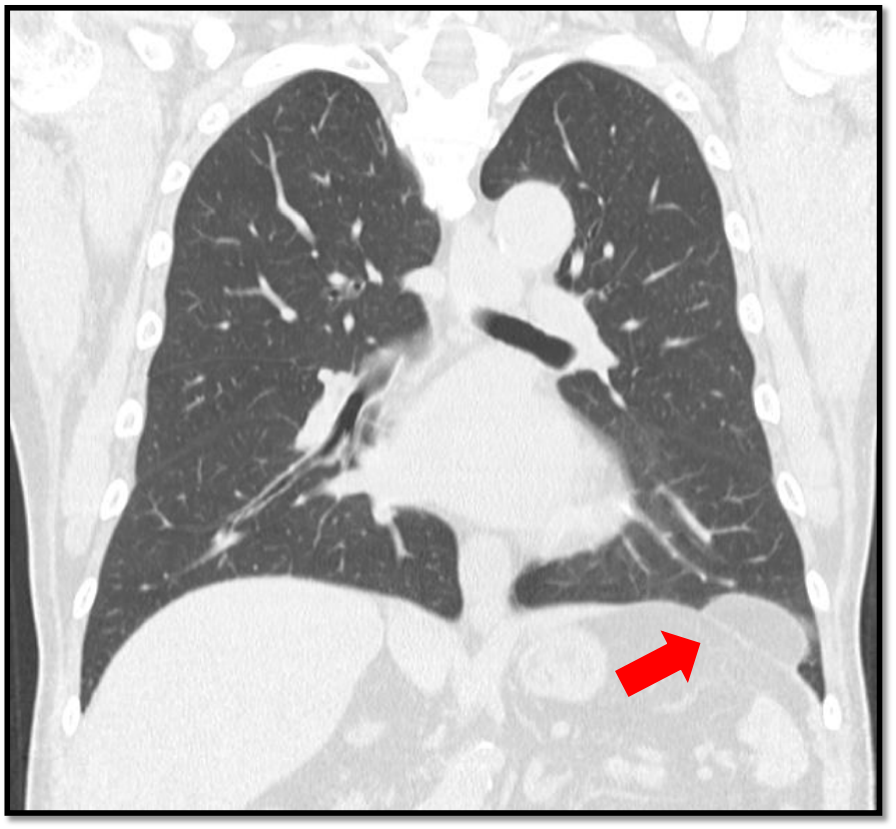
Chest computed tomography (CT) showed a 5-cm solid tumor on the left diaphragm

**Fig. 2:**
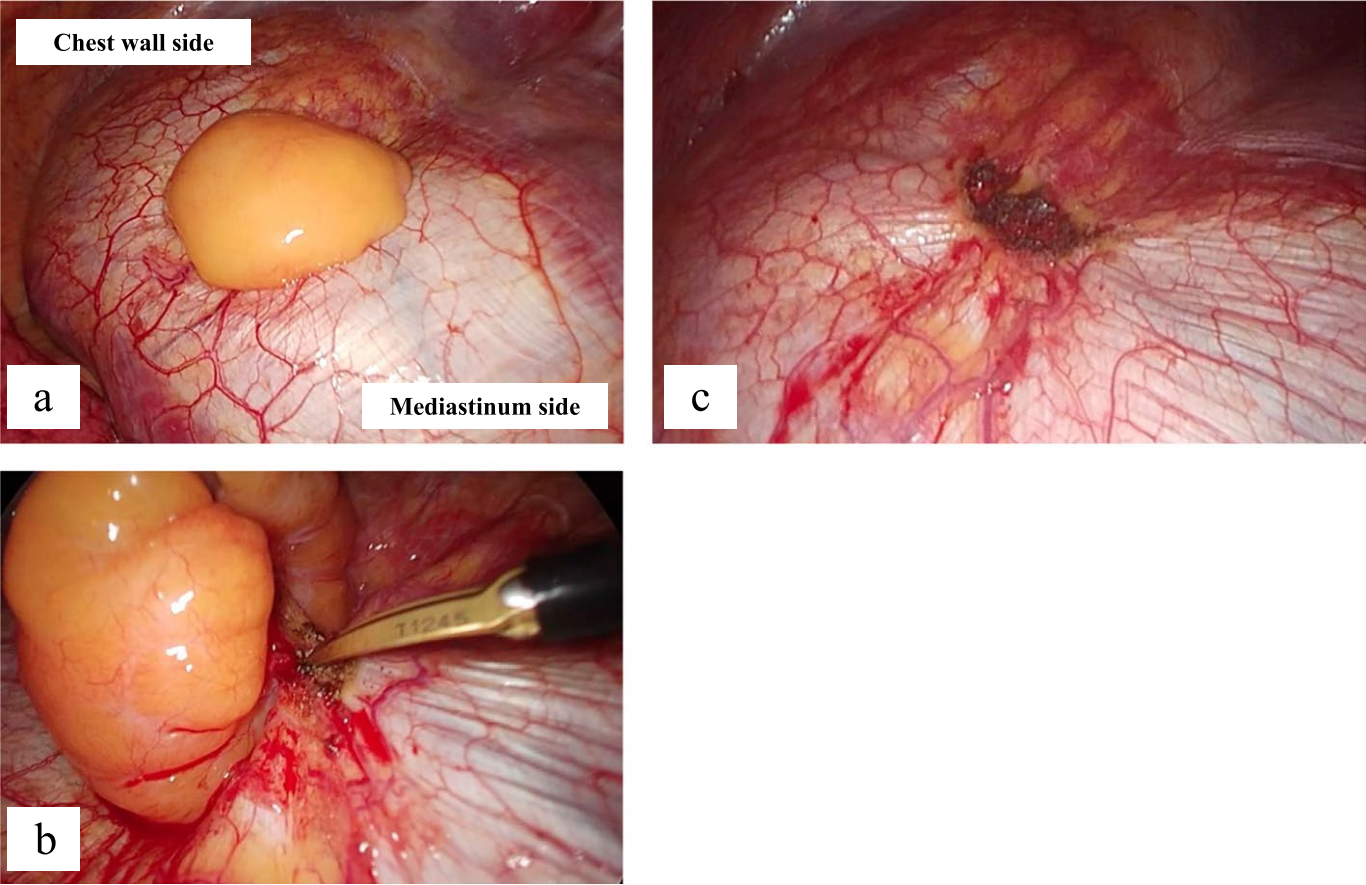
The first operation findings. The tumor was located on the left central tendon of the diaphragm (**a**). We dissected and excised the tumor stalk site and resected this tumor using an electric scalpel (**b**). After resecting the tumor, there was slight heat damage (about 5 × 4 mm in diameter) to the diaphragm (**c**)

Chest computed tomography (CT) showed a 5 × 2-cm solid tumor on the left diaphragm (Fig. 1). He was diagnosed with a tumor originating from the left diaphragm, with lipoma or liposarcoma being the most likely differential diagnosis. Although he was asymptomatic, the tumor might have been malignant. We therefore decided to resect this tumor via three-port video-assisted thoracic surgery (VATS). This tumor was pedunculated at the left central tendon of the diaphragm. We therefore dissected the tumor using an electric scalpel (Fig. 2). Although there was about 5 × 4 mm in diameter heat damage to the diaphragm, it was not reinforced because it was very minor injury. The operation time was 30 min, and there was no active bleeding. He was healthily discharged on postoperative day 6. This tumor’s final pathological diagnosis was lipoma, and margin was free. There was no significant change on an examination 2 months after surgery.

**Fig. 3:**
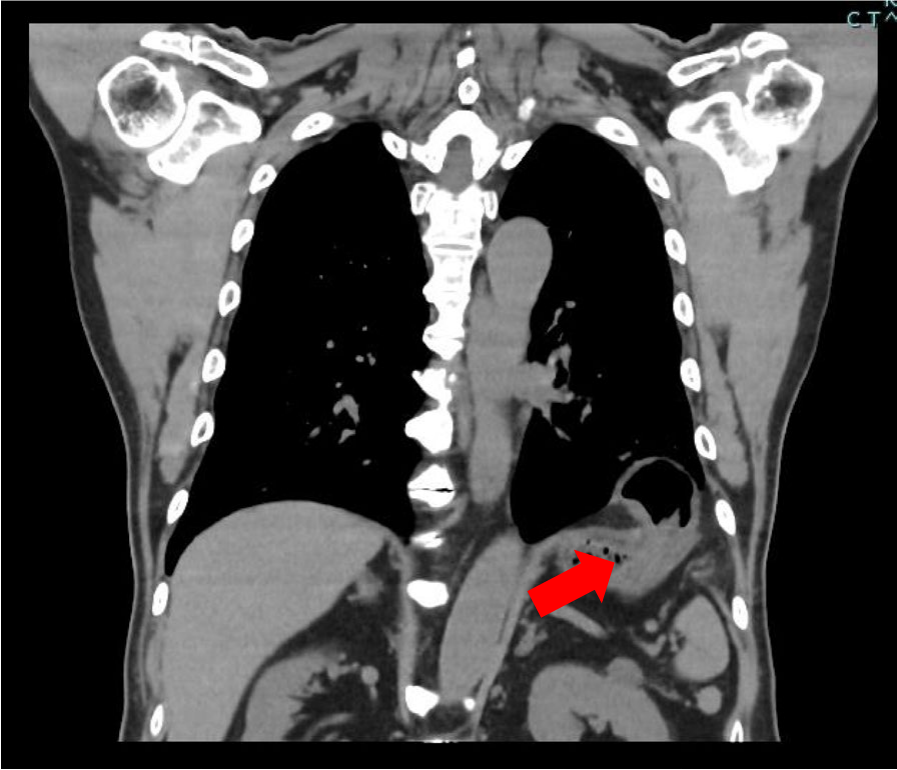
One year after first surgery, we scheduled a routine CT scan. We discovered a left diaphragmatic hernia

He was diagnosed with a left diaphragmatic hernia without any symptoms by routine CT examination which scheduled 1 year after surgery (Fig. 3). Although this patient was asymptomatic, we persuaded him to be hospitalized and planned an operation.

**Fig. 4:**
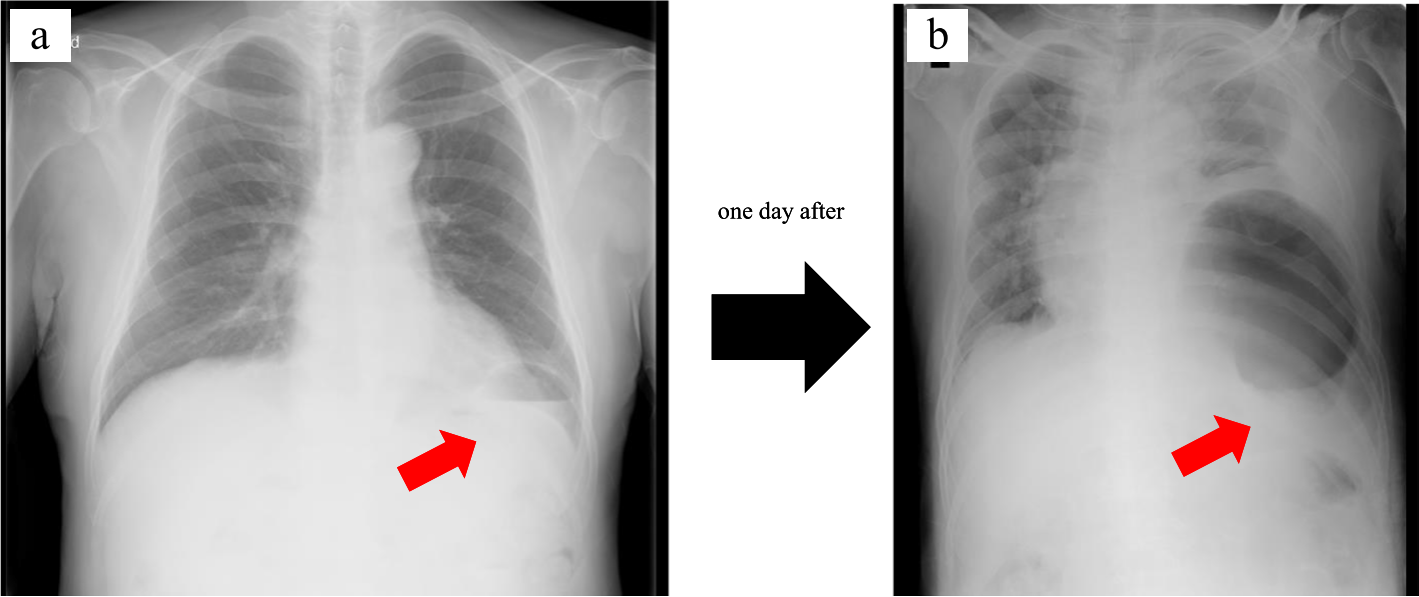
One day after hospitalization, chest X-ray showed acute exacerbation of the left diaphragmatic hernia (**a**, **b**)

**Fig. 5:**
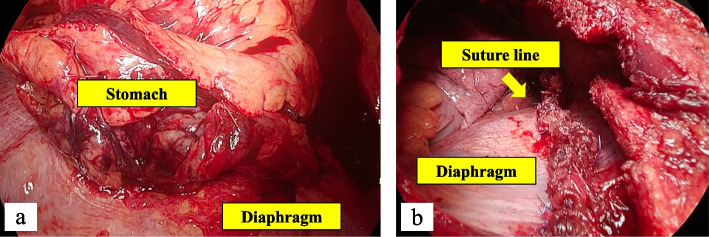
Second operation findings in the thoracic cavity. The stomach was stuck in the hernia and could not be easily removed (**a**). We sutured the left diaphragm and finished the operation (**b**)

One day after hospitalization, on the morning of the operation, he suddenly complained of left back pain. Chest X-ray at that time showed hollow organs expanding in the thoracic cavity, and we diagnosed acute exacerbation of the left diaphragmatic hernia (Fig. 4). We therefore immediately performed emergency surgery via 8th intercostal thoracotomy at first, during which the stomach was found to be incarcerated. Therefore, we added laparotomy. The wound was a continuous incision in the chest and abdomen. About two-thirds of the stomach have moved into the thoracic cavity through the hernia orifice and was incarcerated, so it could not be release easily (Fig. 5a). We therefore cut the diaphragm and freed the stomach. His gastric ischemia also improved with this, and there was no need for gastrectomy. The hernia hole was about 6 × 6 cm. We performed ten horizontal mattress sutures with using pledget followed by an additional running suture. After that, we finished the operation (Fig. 5b). The operation time was 151 min, and operation bleeding was 50 ml. No adverse events or complications were seen, and he was discharged on postoperative day 11. Three months after this surgery, this patient is doing very well.

## Comment

Iatrogenic diaphragmatic hernias have been reported as a rare complication of thoracic and abdominal surgery [1–3, 4]. The low incidence of iatrogenic diaphragmatic hernia might be the result of a high misdiagnosis rate [1]. Despite the rare occurrence of iatrogenic diaphragmatic hernia, the mortality rate following emergency surgery for strangulated or perforated bowel or stomach is between 20 and 80% [6]. To our knowledge, there have been no reports of delayed-onset (1 year) iatrogenic left diaphragmatic hernia after diaphragm pedunculated lipoma resection with minimally invasive surgery (VATS).

This report has three important implications. First, the timing of our regular follow-up after the first surgery with CT was extremely fortunate. As this patient was asymptomatic, the diaphragmatic hernia was only discovered on routine CT 1 year after the first surgery. Three days after this scan, the patient suddenly complained of left-side back pain. Chest X-ray showed left diaphragmatic hernia exacerbation. Since the patient was already hospitalized at that time, we were able to smoothly perform emergency surgery. The surgical findings showed an incarcerated stomach, and we were able to release the hernia incarceration without gastrectomy. If surgery had been delayed, gastrectomy would have been necessary. In this case, the hernia developed 1 year after surgery, so we can recommend that the patient be followed up several times with CT from 1 to 2 years after surgery.

Second, caution is required when using an electric scalpel near the diaphragm. Studies have speculated that the diaphragm could be inadvertently injured intraoperatively through contact with energy devices, such as an electrocautery and ultrasonic coagulation shears, resulting in a delayed diaphragmatic hernia [3, 7]. We think that special attention should be paid to heat damage in manipulations around the diaphragmatic tendon center. Hashimoto et al. reported that the heat generated by soft coagulation might have unexpectedly reached and damaged the deep areas of the diaphragmatic muscle. In addition to the latent heat injury to the diaphragmatic muscle, the pressure gradient between the thoracic and abdominal cavities, which is continuously present within the injured diaphragmatic area, might be a cause of the diaphragmatic hernia [4]. In the present case, we used an electric scalpel in normal coagulation mode on the left-side diaphragm. Therefore, the coagulation area was very narrow and shallow. We also finished the operation without suturing the diaphragm; had we sutured the diaphragm in the first surgery, delayed diaphragmatic hernia might not have occurred.

Third, most delayed diaphragmatic hernias are on the left side. Several reports have found that delayed diaphragmatic hernia on the right side is not unheard of, but most cases do indeed occur on the left side [1–3, 8], as the liver is anatomically located on the right side.

## Conclusions

We reported a case of delayed iatrogenic left-side diaphragmatic hernia after diaphragm pedunculated lipoma resection with minimally invasive surgery. Caution should be exercised when using energy devices on the diaphragmatic surface, especially the left side, to avoid inducing delayed diaphragmatic hernia. Reinforcement of the diaphragm may be necessary, depending on the situation. In cases of surgery involving the left-side diaphragm, it seems that careful follow-up after surgery is necessary.

## Data Availability

Not applicable.

## Abbreviations

CT: Computed tomography
VATS: Video-assisted thoracic surgery
